# Targeting USP14 enhances immunotherapy response by reprogramming tumor-associated macrophages in colon cancer

**DOI:** 10.1016/j.isci.2026.115362

**Published:** 2026-03-13

**Authors:** Dan Xiao, Jun Fang, Hui Jian, Yang Yu

**Affiliations:** 1 Department of Gastroenterology, The Sixth Hospital of Wuhan, Affiliated Hospital of Jianghan University, Wuhan, Hubei Province 430000, China; 2 Department of Gestrointestinal Surgery, The Sixth Hospital of Wuhan, Affiliated Hospital of Jianghan University, Wuhan, Hubei Province 430000, China

**Keywords:** Biochemistry, Cancer, Immunology

## Abstract

Combining immunotherapy with other treatments improves survival in colorectal cancer (CRC), yet some patients remain unresponsive. Tumor-associated macrophages (TAMs) are a key immune cell population driving this immunotherapy resistance and fostering an immunosuppressive microenvironment. To overcome this, we screened a deubiquitinating enzyme (DUB) library targeting TAMs and identified USP14 as specifically upregulated in TAMs. Inhibiting USP14 reversed their pro-tumor functions, promoted M1 polarization, enhanced tumor cell killing, and activated effector T cells. USP14 inhibition also increased PD-L1 expression on tumor cells, alleviating T cell suppression. *In vivo*, combining a USP14 inhibitor with an anti-PD-1 antibody synergistically enhanced immunotherapy efficacy, suppressed tumor progression, and improved survival in a mouse colon cancer model. Thus, USP14 is a promising target to overcome immunotherapy resistance in CRC.

## Highlights

• USP14 upregulation in colon cancer TAMs maintains their M2-like immunosuppressive state
• USP14 inhibition reprograms TAMs to M1-like, boosts CD8^+^ T cells, and immunostimulates TME
• USP14 inhibition induces PD-L1 on tumor cells, providing a rationale for combination with ICB
• USP14/PD-1 dual blockade overcomes immunotherapy resistance in colon cancer

## Graphical abstract

**Figure:**
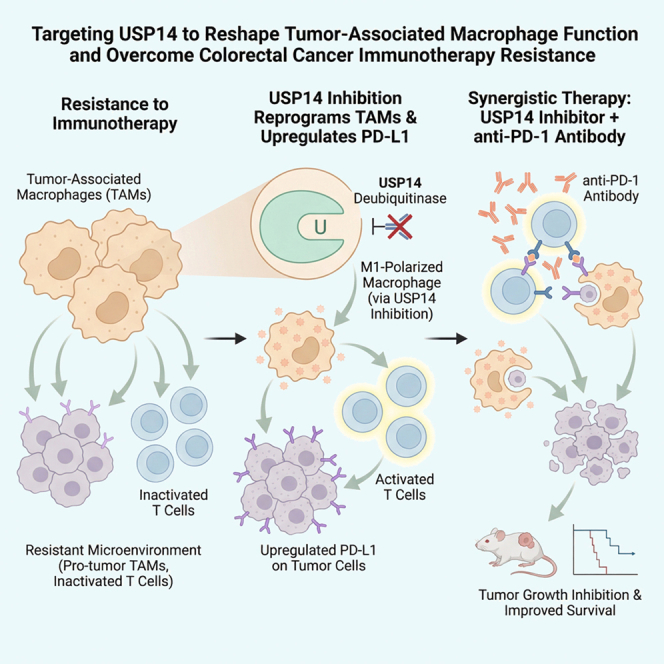

## Introduction

Colorectal cancer (CRC) remains a leading cause of cancer-related mortality worldwide, with its pathogenesis involving a complex interplay of genetic mutations, epigenetic alterations, and microenvironmental dysregulation.^1^^,^^2^ While conventional modalities such as surgery, chemotherapy, and radiotherapy form the cornerstone of treatment, a significant proportion of patients develop resistance or exhibit intrinsic insensitivity, underscoring the urgent need for novel therapeutic strategies.^3^^,^^4^ In recent years, immunotherapy, particularly immune checkpoint blockade (ICB) targeting pathways such as PD-1/PD-L1 and CTLA-4, has revolutionized oncology and shown promise in subsets of patients with CRC.^5^^,^^6^ However, the overall response rate to ICB in CRC, especially in microsatellite stable (MSS) tumors, which constitute the majority of cases, remains disappointingly low.^7^^,^^8^ A primary barrier to effective immunotherapy is the immunosuppressive tumor microenvironment (TME), a dynamic ecosystem that actively subdues anti-tumor immunity.^9^

Within this suppressive TME, tumor-associated macrophages (TAMs) have emerged as pivotal orchestrators of immune evasion and therapeutic resistance.^10^^,^^11^ Derived from circulating monocytes, TAMs exhibit remarkable plasticity, polarizing toward a spectrum of functional states largely categorized into pro-inflammatory, anti-tumor M1-like phenotypes and anti-inflammatory, pro-tumor M2-like phenotypes.^12^ In CRC, tumor-derived signals such as CSF-1, IL-4, and IL-10 drive the recruitment and polarization of monocytes into M2-like TAMs, which in turn promote tumor progression, angiogenesis, matrix remodeling, and suppression of effector T cell functions.^13^^,^^14^ Given their abundance—often constituting over 50% of the leukocyte infiltrate—and their central role in maintaining immunosuppression, TAMs represent a highly attractive yet challenging therapeutic target.^15^^,^^16^ Current strategies to modulate TAMs, including depletion, inhibition of recruitment, or phenotypic reprogramming, often face limitations such as lack of specificity, systemic toxicity, or insufficient clinical efficacy.^17^^,^^18^ Therefore, identifying novel, precise molecular targets within TAMs to safely reprogram their function from a pro-tumor to an anti-tumor state is a critical unmet need in CRC immunotherapy.

The ubiquitin-proteasome system is a fundamental regulator of cellular protein homeostasis, signal transduction, and immune responses. Deubiquitinating enzymes (DUBs), which reverse protein ubiquitination, have gained recognition as key players in cancer biology and are emerging as promising therapeutic targets.^19^ Several DUBs, including USP1, USP7, and USP14, have been implicated in tumor cell proliferation, survival, and drug resistance across various cancer types.^20^^,^^21^^,^^22^ Intriguingly, a growing body of evidence suggests that DUBs also play crucial roles in regulating immune cell functions. For instance, USP4 and USP19 have been linked to modulating inflammatory signaling pathways in macrophages.^23^^,^^24^ However, the landscape of DUBs specifically governing the functional polarization and immunosuppressive activity of TAMs within the CRC TME remains largely unexplored. Systematic investigation in this area could unveil novel regulatory nodes for therapeutic intervention.

Among DUBs, ubiquitin-specific protease 7 (USP7) has been implicated in reprogramming TAMs in lung cancer, highlighting its role in modulating the tumor immune microenvironment.^25^ Inspired by this, we sought to investigate whether specific DUBs similarly regulate TAM reprogramming in CRC. Alongside USP7, Ubiquitin-Specific Protease 14 (USP14) has also drawn attention for its oncogenic roles. It stabilizes key proteins, such as IDO1, in CRC to promote immunosuppression, regulates the TAZ oncogene in pancreatic cancer, and is associated with therapy resistance in other malignancies.^26^^,^^27^^,^^28^^,^^29^^,^^30^ Despite these advances, its function within the immune compartment, particularly in TAMs, remains unknown. We hypothesized that USP14 might be a critical regulator of TAM polarization in CRC. Herein, we conducted a systematic screen to identify DUBs dysregulated in CRC-derived TAMs. We identified USP14 as a specifically upregulated DUB in TAMs and functionally characterized its role in regulating macrophage polarization. Furthermore, we investigated the therapeutic potential of pharmacologically inhibiting USP14, both as a monotherapy and in combination with anti-PD-1 blockade, for the treatment of colon cancer. Our findings position USP14 as a novel myeloid-specific target to reprogram the TME and overcome immunotherapy resistance in CRC.

## Results

### USP14 is specifically upregulated in colon cancer TAMs and regulates their M2 phenotype *in vitro*

**Figure 1:**
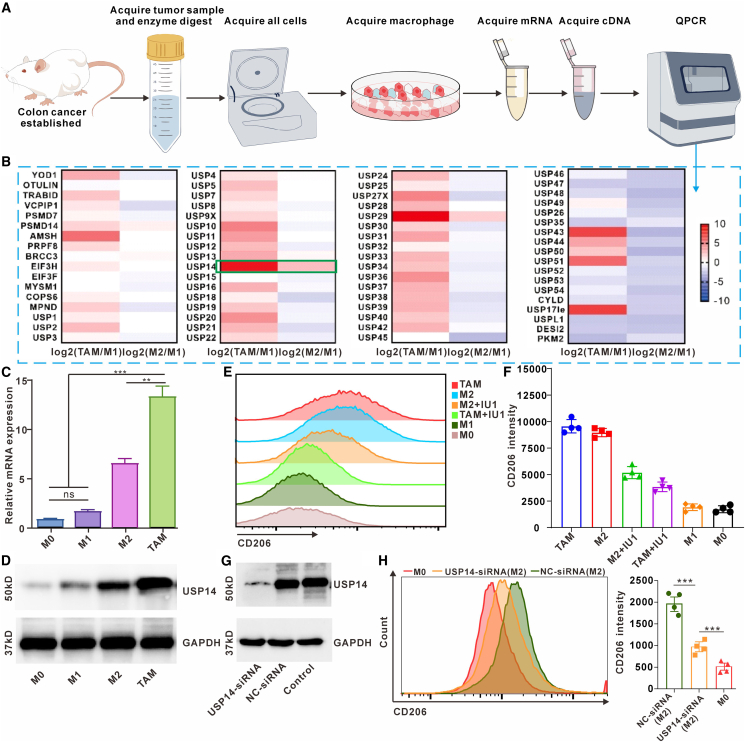
Targeting USP14 inhibits murine colon cancer TAMs' M2 phenotype and function *in vitro* (A) Schematic diagram of the experimental procedure of TAMs acquisition in colon cancer. (B) The mRNA expression of common genes related to DUBs was detected by RT-PCR in TAMs, M1 (LPS-stimulated M1), and M2 (IL-4-stimulated M2) induced from borrow-derived macrophage. (C) Statistics of the mRNA expression of USP14 in TAMs isolated from colon cancer, M0, M1, and M2 derived from BMDMs by using the RT-PCR method (*n* = 3 repeats). (D) Western blotting shows the expression of USP14 in TAMs, M0, M1, and M2 induced from BMDMs. (E and F) Flow cytometry analyses of the expression of CD206 in TAMs, M0, M1, and M2 induced from BMDMs, which had been treated with IU1 (5 μM) for 24 h. Data are presented as the mean ± SEM (*n* = 4). (G) Detection of the expression of USP14 in TAMs, which were transfected with either NC- or USP14-siRNA, by western blotting. (H) The expression of CD206 in M2 macrophages treated with NC-siRNA or USP14-siRNA group was detected and statistics by flow cytometry. Data are presented as the mean ± SEM. ∗*p* < 0.05, ∗∗*p* < 0.01, and ∗∗∗*p* < 0.001, and ns: not significant.

To identify DUBs that may govern TAM function in the immunosuppressive colon cancer microenvironment, we first established a syngeneic mouse model by subcutaneously implanting MC38 colon adenocarcinoma (COAD) cells into C57BL/6 mice. TAMs (CD11b^+^F4/80^+^) were subsequently isolated from harvested tumors. In parallel, bone marrow-derived macrophages (BMDMs) were polarized *in vitro* into classically activated M1 macrophages (using LPS) or alternatively activated M2 macrophages (using IL-4 and IL-13). We then performed qPCR screening targeting a panel of 68 common DUB-related genes across these three macrophage populations (TAMs, M1-BMDMs, M2-BMDMs). This analysis revealed a distinct expression profile in TAMs. Specifically, four DUBs—USP14, USP29, USP43, and USP17—showed significantly higher mRNA expression in TAMs compared to both M1 and M2 BMDMs (Figure 1B). Among these, only USP14 has a well-characterized, commercially available small-molecule inhibitor (IU1), making it a tractable target for immediate functional and therapeutic investigation.^31^ Therefore, we focused subsequent studies on USP14.

We confirmed the screening results using independent assays. Quantitative PCR analysis demonstrated that USP14 mRNA levels were approximately 12.8-fold higher in TAMs than in naive (M0) BMDMs, 6.2-fold higher than in M1-BMDMs, and 1.8-fold higher than in M2-BMDMs (Figure 1C). Western blot analysis corroborated these findings at the protein level, showing markedly increased USP14 protein expression specifically in TAMs (Figure 1D).

To determine whether USP14 activity is functionally linked to the M2 phenotype, we treated both isolated TAMs and IL-4/IL-13-induced M2-BMDMs with the USP14 inhibitor IU1 (10 μM) for 24 h. Flow cytometry analysis of the canonical M2 marker CD206 revealed that IU1 treatment significantly reduced the expression of CD206. In TAMs, the fluorescent intensity of CD206 decreased 2-fold compared with the IU1-treated group. Similarly, in M2-BMDMs, IU1 treatment reduced 1.8-fold compared with the IU1-treated group (Figures 1E and 1F). To establish a direct causal relationship, we performed loss-of-function experiments using siRNA. Transfection of M2 macrophage with USP14-specific siRNA achieved a knockdown efficiency of >80% at the protein level compared to non-targeting control (NC) siRNA (Figure 1G). This genetic ablation of USP14 resulted in a significant decrease in the CD206 expression (Figure 1H). Collectively, these *in vitro* data demonstrate that USP14 is selectively overexpressed in colon cancer TAMs and is a critical regulator of their M2-polarized state.

### High USP14 expression in human colon adenocarcinoma correlates with an immunosuppressive tumor microenvironment

**Figure 2:**
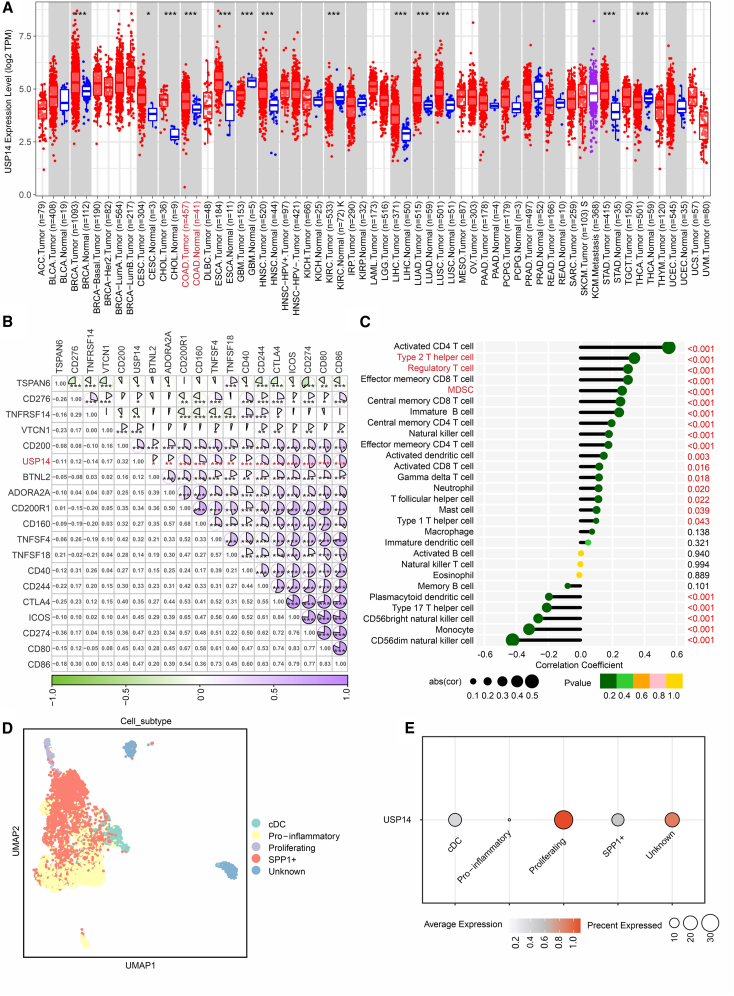
USP14 expression in COAD is negatively correlated with anti-tumor immunity in TCGA (A) USP14 mRNA expression levels in various human cancers (red) and normal tissues (blue) from the TCGA database, showing elevated expression in COAD compared to normal colon and many other tumor types. (B) Correlation of USP14 with the expression of immunomodulatory molecules from the TCGA database. (C) Relationship between USP14 expression and immune cell infiltration from the TCGA database. (D) Single-cell sequence to identify cell type in COAD microenvironment (data from GSE132465). (E) Violin plot depicts the relative expression distribution of USP14 across the major cell clusters identified in the single-cell RNA-seq data (GSE132465) from colon cancer tissues. Among them, the cells with the highest expression level of proliferation were used as the internal reference for relative expression analysis.

To translate our murine findings to human disease, we analyzed clinical data from The Cancer Genome Atlas (TCGA) COAD cohort. USP14 mRNA expression was significantly elevated in COAD tumor tissues (*n* = 471) compared to matched adjacent normal tissues (*n* = 41) and was notably elevated relative to many other cancer types in the pan-cancer analysis (Figure 2A). This supports its potential role as an oncoprotein in human CRC. We next investigated the correlation between USP14 expression and key immunomodulatory molecules. USP14 levels showed a significant negative correlation with the expression of several immunomodulatory molecules, including PD-L1 (CD274) and CTLA-4, while correlating positively with CD276 (B7-H3) (Figure 2B). Furthermore, analysis using immune cell deconvolution algorithms (CIBERSORT) revealed that high USP14 expression was associated with the increased infiltration of immunosuppressive cell types, such as M2 macrophages, regulatory T cells, and myeloid-derived suppressor cells (MDSCs) (Figure 2C).

To validate this at a single-cell resolution, we analyzed a public single-cell RNA-seq dataset (GSE132465) from human CRC tissues.^32^ Unsupervised clustering identified major cell populations within the TME (Figure 2D). The expression of USP14 was predominantly enriched in macrophages and a subset of proliferating cells (Figure 2E). These bioinformatic analyses consistently indicate that elevated USP14 in human COAD is associated with an immunosuppressive TME characterized by altered checkpoint molecule expression and enriched inhibitory leukocyte infiltration.

### Pharmacological inhibition of USP14 suppresses tumor growth and remodels the TME toward an immunostimulatory state *in vivo*

**Figure 3:**
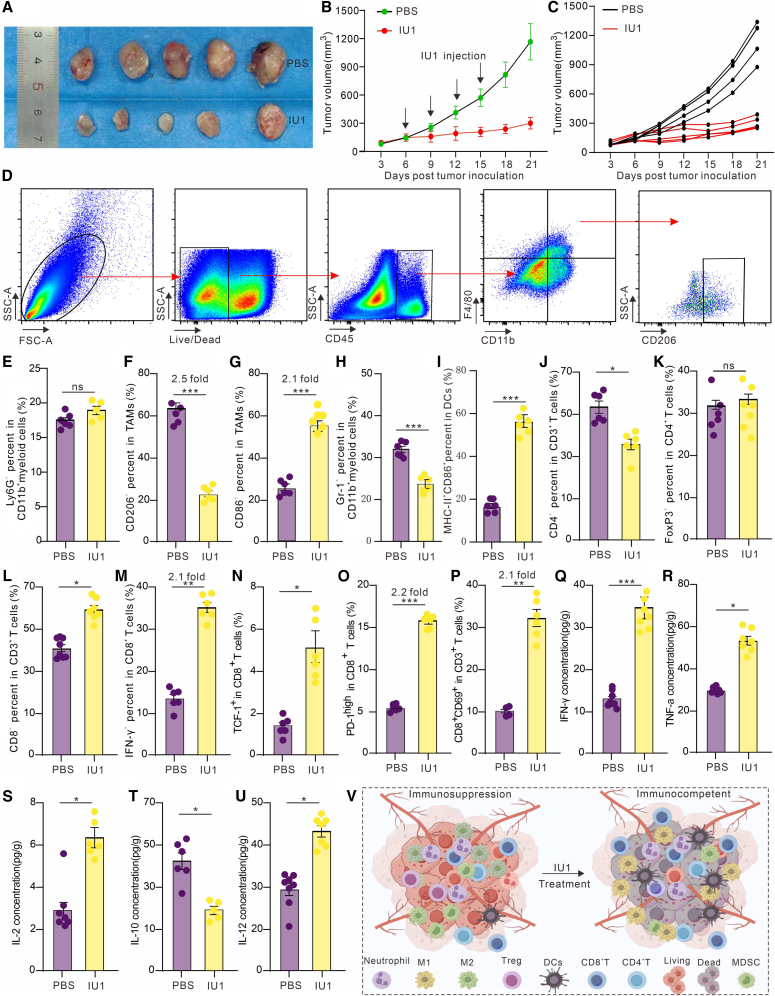
Targeting USP14 inhibits tumor growth and induces local anti-tumor immunity *in vivo* (A) Representative images of MC38 tumors harvested on day 21 post-inoculation from mice treated with IU1 (20 mg/kg, i.p., on days 6, 9, 12, and 15) or vehicle control. (B) Statistics of MC38 tumor growth rate following IU1 or vehicle treatment *in vivo*. The intraperitoneal injection dose of IU1 was 20 mg/kg, administered on days 6, 9, 12, and 15. Data are presented as the mean ± SEM (*n* = 6 per group). (C) Spider diagram of the tumor volume growth in each mouse from the IU1 group and the PBS group. (D) Gating strategy for the detection of the TAMs by flow cytometry. We first obtained live cells, and then identified cells that were positive for CD45, CD11b, F4-80, and CD206 as M2 macrophages. (E–P) Proportions of neutrophil (E), M2 macrophage (F), M1 macrophage (G), MDSC (H), activated DCs (I), CD4 T cell (J), Treg cells (K), CD8 T cell (L), IFN-γ^+^ CD8 T cell (M), precursor exhausted T cells (TCF-1^+^) (N), effective CD8 T cells (PD-1^+^) (O) and activated CD8 T cell (CD69^+^) (P) in the TME of the IU1 group and the control group by using flow cytometry. (Q–U) Cytokines IFN-γ (Q), TNF-α (R), IL-2 (S), IL-10 (T), and IL-12 (U) in the TME of each group were detected by Mul-Analyte Flow Assay Kit. (V) Schematic illustration of the proposed mechanism of action of IU1 in reprogramming the tumor microenvironment. Data are presented as the mean ± SEM. ∗*p* < 0.05, ∗∗*p* < 0.01, and ∗∗∗*p* < 0.001, and ns: not significant.

Given the strong *in vitro* and clinical correlations, we evaluated the therapeutic potential of USP14 inhibition *in vivo*. MC38 tumor-bearing mice were treated intraperitoneally with the USP14 inhibitor IU1 (20 mg/kg) or vehicle every three days, starting from day 6 post-tumor inoculation. IU1 treatment potently inhibited tumor growth. By day 21, the average tumor volume in the IU1 group was 235 ± 35 mm^3^, compared to 1100 ± 98 mm^3^ in the vehicle group, representing an inhibition rate of 78.0% (Figures 3A–3C).

To elucidate the immunologic mechanisms underlying the antitumor effect of USP14 inhibition, we performed a comprehensive immunophenotyping analysis of the TME on day 12 post-treatment initiation. We first assessed the impact of IU1 on innate immune cell populations. Flow cytometric analysis, with the gating strategy for TAMs detailed in Figure 3D, revealed that IU1 treatment induced a profound reprogramming of the suppressive myeloid compartment. The frequency of M2-polarized TAMs, identified as live CD45^+^CD11b^+^F4/80^+^CD206^+^ cells, was dramatically reduced by approximately 2.5-fold (Figure 3F). Concordantly, the proportion of M1-polarized macrophages (CD11b^+^F4/80^+^CD86^+^) showed a significant increase, indicating a shift in the TAM polarization balance toward a pro-inflammatory phenotype (Figure 3G). Furthermore, IU1 treatment significantly reduced the infiltration of MDSCs (identified as CD11b^+^Gr-1^+^), another key population contributing to immunosuppression (Figure 3H). In contrast, the proportion of neutrophils (CD11b^+^Ly6G^+^) remained statistically unchanged compared to controls (Figure 3E).

Subsequently, we evaluated the effects on adaptive immunity, particularly T cell populations. While the overall infiltration of CD4^+^ T helper cells exhibited a modest but significant decrease (Figure 3J), the frequency of regulatory T cells (Tregs, CD4^+^Foxp3^+^) was unaltered (Figure 3K). Most strikingly, IU1 treatment led to a substantial expansion and functional activation of the CD8^+^ cytotoxic T lymphocyte (CTL) compartment. The total frequency of CD8^+^ T cells increased by 1.5-fold (Figure 3L). More importantly, critical functional subsets within this population were significantly augmented: IFN-γ-producing CTLs increased by 2.7-fold (Figure 3M), activated CD8^+^ T cells (CD69^+^) increased by 3.1-fold (Figure 3P), antigen-experienced cells (PD-1^+^) increased by 3.1-fold (Figure 3O), and precursor exhausted-like T cells (TCF-1+) increased by 2.2-fold (Figure 3N).

Consistent with this cellular immune activation, cytokine profiling of tumor lysates revealed a shift toward a pro-inflammatory milieu. Levels of IFN-γ, TNF-α, IL-2, and IL-12 were significantly elevated in the IU1-treated group, while the level of immunosuppressive IL-10 was reduced by 50% (Figures 3Q–3U). These data demonstrate that USP14 inhibition *in vivo* not only reprograms TAMs away from an M2 phenotype but also orchestrates a multifaceted activation of CD8^+^ T cell-mediated anti-tumor immunity.

### The antitumor efficacy and T cell activation induced by IU1 are dependent on macrophage reprogramming

**Figure 4:**
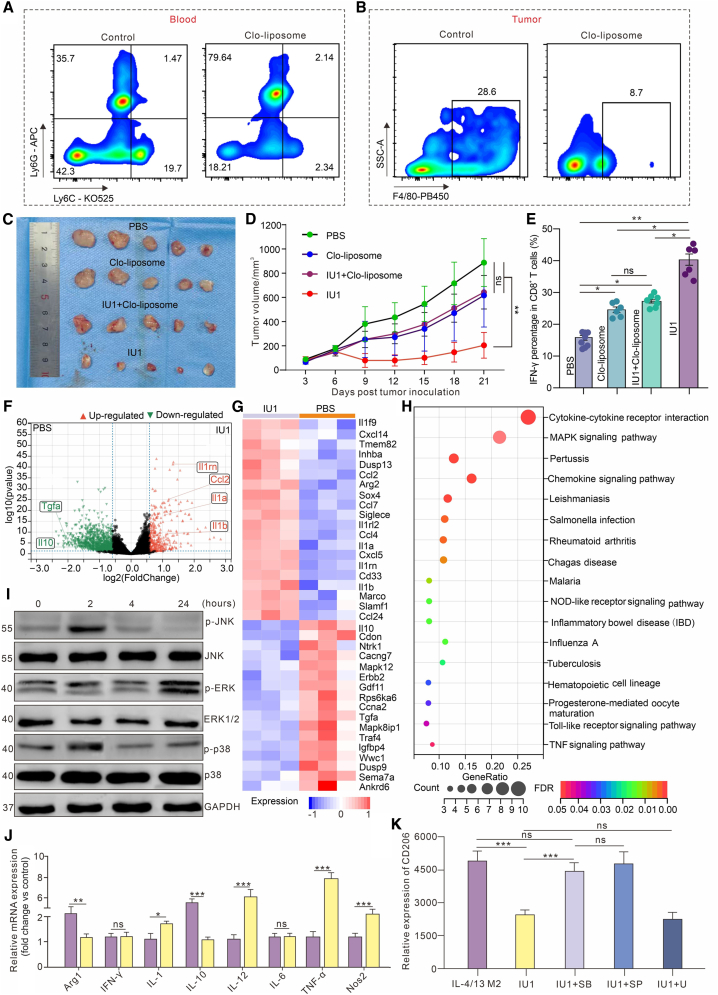
Identification of the mechanism underlying IU1-mediated reprogramming of M2 macrophages (A and B) Verification of the efficiency of *i.v.* injection of clodronate liposome (Clo) in depleting TAMs of the blood (A) and the TME (B). (C and D) Statistics of tumor size monitored for 21 days in MC38 tumor-bearing mice after various indicated treatments. (E) Statistic of the percentage of CTLs in the TME after the indicated treatment. (F) Volcano plots of the differentially expressed genes between the PBS and the IU1 group. Red dots show significantly up-regulated genes in the IU1 group, and green dots show significantly down-regulated genes. (G) Heatmap illustrates the differentially expressed M1-and M2-related genes in TAMs in the IU1 group and the PBS group based on RNA sequencing results. (H) KEGG analysis identifies the 17 most enriched pathways based on the differentially expressed genes of the two groups. (I) Western blotting of *p*-JNK, *p*-ERK, p-p38, and GAPDH in IL-4/13-BMDM M2 cells treated with IU1 at the indicated time points. (J) RT-PCR to verify the typical M1/M2 polarization-related genes in M2 macrophages after treatment with IU1. (K) Flow cytometry analysis of CD206 expression on the IL-4/13-induced BMDM M2 cells from various indicated treatments. Treatments indicated: DMSO stimulation, IU1 (10 μM) stimulation, IU1 (10 μM) stimulation in the presence of inhibitors of p38 (SB203580, 10 μM), JNK (SP600125, 10 μM), Erk1/2 (U0126-EtOH, 10 μM). Statistical analysis was performed using one-way ANOVA with Tukey’s multiple comparison test. Data are presented as the mean ± SEM. ∗*p* < 0.05, ∗∗*p* < 0.01, and ∗∗∗*p* < 0.001, and ns: not significant.

To formally test whether TAMs are the primary cellular target mediating IU1’s effects *in vivo*, we employed a macrophage depletion strategy using clodronate liposomes. Administration of clodronate liposomes effectively depleted macrophages, reducing CD11b+F4/80+ cells in the blood by >85% and in tumors by >70% (Figures 4A and 4B). While IU1 monotherapy again showed strong tumor suppression (68% inhibition), this effect was partially but significantly reversed in mice receiving both clodronate and IU1, where the tumor inhibition rate dropped to 32% (Figures 4C and 4D). The residual activity suggests that IU1 may have additional, direct effects on tumor cells, consistent with previous reports. Crucially, the robust infiltration of IFN-γ+ CTLs induced by IU1 treatment was completely abrogated in macrophage-depleted mice (Figure 4E). This result provides direct evidence that the immunostimulatory effects of USP14 inhibition, particularly the activation and recruitment of effector CD8^+^ T cells, are contingent upon its action on TAMs.

### USP14 inhibition promotes M1 polarization via activation of the MAPK signaling pathway

To elucidate the molecular mechanism by which USP14 inhibition reprograms macrophages, we performed RNA sequencing on sorted TAMs from IU1- versus vehicle-treated tumors. Differential expression analysis identified 1,247 upregulated and 986 downregulated genes (fold change >2, *p* < 0.05) (Figure 4F). Gene set enrichment analysis confirmed a clear shift: M1-associated gene signatures (Tnf, Il12b, Nos2) were upregulated, while M2-associated signatures (Arg1, Mrc1, Cd163) were downregulated (Figure 4G). Kyoto Encyclopedia of Genes and Genomes (KEGG) pathway analysis of the differentially expressed genes identified the MAPK signaling pathway as the most significantly enriched (Figure 4H).

We therefore investigated the role of MAPK signaling. Western blot analysis of IL-4/13-induced M2-BMDMs treated with IU1 revealed a time-dependent increase in the phosphorylation of key MAPK components: JNK, ERK, and p38, with peak activation observed at 2 h post-treatment (Figure 4I). qPCR validation confirmed that IU1 treatment in M2-BMDMs significantly increased mRNA levels of Tnf (4.1-fold) and Il12b (3.5-fold), while decreasing Arg1 (70% reduction) and IL-10 (65% reduction) (Figure 4J). To functionally link MAPK activation to the phenotypic switch, we co-treated M2-BMDMs with IU1 and specific pharmacological inhibitors of p38 (SB203580), JNK (SP600125), or ERK (U0126). Flow cytometry analysis showed that the inhibition of p38 or JNK significantly restored CD206 expression that had been suppressed by IU1, whereas ERK inhibition resulted in only a modest and non-significant reversal (Figure 4K). This suggests that JNK and p38 may play more dominant roles in mediating the repolarizing effect of USP14 inhibition, consistent with previous studies implicating these pathways in M2-to-M1 phenotypic switching.^25^ The precise upstream substrate(s) through which USP14 regulates MAPK activation in macrophages remain to be identified.

### Combined blockade of USP14 and PD-1 exerts synergistic anti-tumor effects *in vivo*

**Figure 5:**
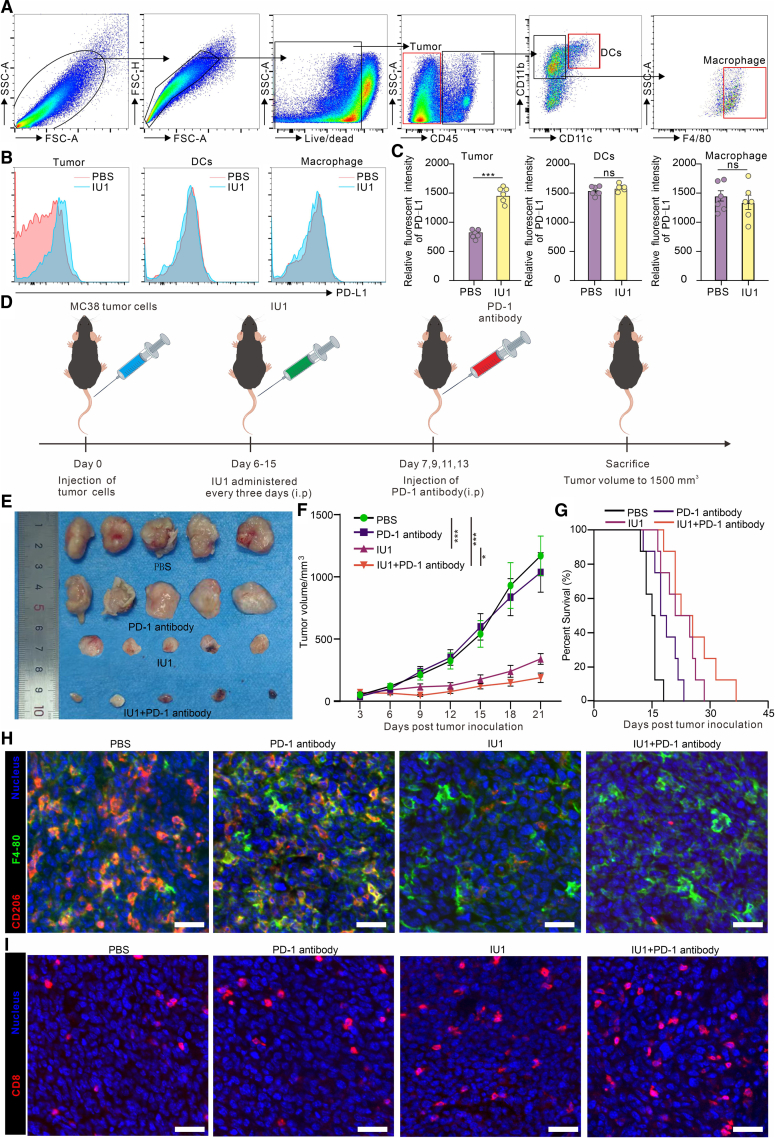
Combined blockade of USP14 and PD-1 exerts a synergistic anti-tumor effect *in vivo* (A) Gating strategy for distinguishing tumor cells, DC cells, and macrophages. (B) Flow cytometry was used to analyze the differences in PD-L1 expression in tumor cells, DC cells, and macrophages in the indicated treatment groups. (C) Flow cytometry was used to statistically analyze the differences in PD-L1 expression in tumor cells, DC cells, and macrophages in the specified groups. (D) Treatment schedule of the combination of IU1 and PD-1 antibody for the MC38 tumor-bearing mice. The intraperitoneal injection dose of IU1 was 20 mg/kg, administered on days 6, 9, 12, and 15. The intraperitoneal injection dose of anti-PD-1 antibody was 7.5 mg/kg, administered on days 7, 9, 11, and 13. (E and F) Tumor growth was monitored for 21 days in MC38 tumor-bearing mice after various indicated treatments. (G) Kaplan-Meier survival plot shows the survival of mice after the indicated treatments (*n* = 10). (H–I) Multicolor immunofluorescence detection of M2 macrophage (H) and CTLs (I) in the TME of the indicated treatment group. Scale bar is 100 μm. Data are presented as the mean ± SEM. ∗*p* < 0.05, ∗∗*p* < 0.01, and ∗∗∗*p* < 0.001, and ns: not significant.

Having established that USP14 inhibition reprograms TAMs and activates anti-tumor immunity, we sought to investigate its impact on the PD-1/PD-L1 axis, a central pathway in immune evasion. Flow cytometric analysis of the TME revealed a cell-type-specific effect of IU1 treatment on PD-L1 expression. While PD-L1 levels on dendritic cells (CD11c+) and macrophages (CD11b+F4/80+) remained largely unchanged, we observed a significant and specific upregulation of PD-L1 on MC38 tumor cells themselves following IU1 treatment (Figures 5A–5C). This finding suggests that while IU1 alleviates immunosuppression by reprogramming TAMs, it may concurrently induce a compensatory adaptive resistance mechanism in tumor cells via enhanced PD-L1 expression.

This observation provided a strong rationale for combining USP14 inhibition with PD-1 blockade. We hypothesized that the IU1-induced increase in tumor cell PD-L1 could be therapeutically exploited by anti-PD-1 antibodies to further unleash T cell activity. To test this, MC38 tumor-bearing mice were randomized into four treatment groups: vehicle control, IU1 monotherapy, anti-PD-1 monotherapy, and the IU1 + anti-PD-1 combination (Figure 5D). Consistent with our hypothesis, the combination therapy demonstrated superior efficacy. While both monotherapies showed moderate anti-tumor activity, their combination resulted in significantly enhanced tumor growth suppression (Figures 5E and 5F). On day 21, the average tumor volume in the combination group was reduced by approximately 90% compared to the control group, significantly outperforming either single agent alone. This synergistic effect translated into a profound survival benefit. Kaplan-Meier analysis showed that the combination group achieved the longest median survival, with 60% of mice surviving beyond day 50, whereas all mice in the control group succumbed by day 30 (Figure 5G). Immunofluorescence analysis of tumor sections provided mechanistic insight into this synergy. The combination therapy group exhibited the most dramatic remodeling of the TME: a near-complete absence of M2 macrophages (F4/80^+^CD206^+^) was accompanied by the most extensive infiltration of CD8^+^ cytotoxic T lymphocytes (Figures 5H and 5I).

In summary, these results demonstrate a critical sequential logic: USP14 inhibition by IU1 reprograms TAMs to activate T cells but also provokes an adaptive increase in tumor cell PD-L1. This induced PD-L1 expression, rather than undermining therapy, creates a specific vulnerability that can be effectively targeted with PD-1 blockade. The consequent combination strategy yields potent synergistic anti-tumor activity by simultaneously dismantling myeloid-mediated suppression (via TAM reprogramming) and overcoming the resulting T cell intrinsic inhibition (via PD-1 blockade).

## Discussion

The immunosuppressive TME is a major determinant of immunotherapy failure in solid tumors, including CRC. Within this milieu, TAMs are not merely bystanders but active contributors to immune suppression, making them a focal point for therapeutic modulation.^10^ However, translating TAM-targeting strategies into clinical success has been challenging due to a lack of specificity and actionable molecular targets. Our study addresses this gap by identifying the DUB USP14 as a novel, functionally critical regulator of M2-like TAM polarization in colon cancer and demonstrating the therapeutic efficacy of its inhibition. Although Gubat et al. have shown that USP14 can promote the occurrence and development of colon cancer, in this study, we clarified that USP14 can reprogram TAMs in the colon cancer microenvironment.^33^

We initiated our investigation with an unbiased screen, which revealed that USP14 is selectively overexpressed in TAMs isolated from murine colon tumors compared to *in vitro*-derived M1 or M2 macrophages. This specific enrichment within the tumor context suggests that USP14 expression is driven by cues from the CRC microenvironment, positioning it as a potential target for tumor-selective immune modulation. Importantly, analysis of human TCGA data corroborated our preclinical finding, showing elevated USP14 expression in COAD tissues compared to normal controls. Furthermore, high USP14 expression correlated with signatures of immunosuppression, including increased infiltration of M2 macrophages and Tregs, and altered expression of checkpoint molecules. These clinical correlations strongly support the translational relevance of USP14 in human CRC pathophysiology.

Functionally, we demonstrated that genetic knockdown or pharmacological inhibition of USP14 effectively reversed the M2 phenotype, as evidenced by decreased expression of CD206. This *in vitro* reprogramming translated into significant *in vivo* antitumor effects. Treatment with the USP14 inhibitor IU1 markedly suppressed MC38 tumor growth, which was accompanied by a profound reshaping of the TME. The myeloid compartment shifted from a suppressive state—characterized by reduced M2-TAMs and MDSCs—towards a more immunostimulatory one. This myeloid reprogramming created a permissive environment for adaptive immunity, leading to a substantial expansion and functional activation of CD8^+^ T cells, including increased frequencies of IFN-γ^+^ CTLs and activated T cell subsets.

A pivotal and clinically insightful finding was the cell-specific effect of IU1 on PD-L1 expression. While it reprogrammed macrophages, it concurrently upregulated PD-L1 specifically on tumor cells. This increase in tumor cell PD-L1 expression may represent an adaptive response to the emerging anti-tumor immune activity.^34^ Indeed, IU1 treatment significantly increased the frequency of IFN-γ-producing CD8^+^ T cells (Figure 3M), and IFN-γ is a well-known inducer of PD-L1 expression in tumor cells. Thus, it is plausible that the observed PD-L1 upregulation is driven by T cell-derived IFN-γ, although direct causality was not tested in this study. Rather than being a drawback, this effect revealed a rational combinatorial opportunity. The IU1-induced PD-L1 upregulation essentially “flags” the tumor cells, making them more susceptible to PD-1/PD-L1 axis blockade. As hypothesized, the combination of IU1 with an anti-PD-1 antibody yielded synergistic antitumor activity and survival benefit, significantly outperforming either monotherapy. This sequence—myeloid reprogramming inducing adaptive resistance that is then specifically blocked—presents a sophisticated two-pronged strategy to comprehensively overcome immune suppression.

Mechanistically, our RNA-seq and pathway analysis revealed that USP14 inhibition is associated with the activation of the MAPK signaling pathways (JNK, ERK, p38) in macrophages. While the MAPK cascade is a well-established regulator of inflammatory gene expression and macrophage polarization,^35^ the direct molecular target of USP14 upstream of these kinases remains undefined. Future studies aimed at identifying ubiquitinated substrates of USP14 in macrophages will be necessary to fully elucidate the mechanistic basis of its immunomodulatory function. Rescue experiments using specific MAPK inhibitors confirmed that this activation is functionally required for the IU1-mediated reduction of the M2 marker CD206. While the precise upstream substrate(s) of USP14 in this pathway remain to be fully elucidated, our data establish a novel link between USP14 activity, MAPK signaling, and the transcriptional program controlling macrophage fate in the TME.

Our study has several limitations. First, while clodronate-mediated macrophage depletion confirmed TAMs as a major cellular target, the partial reversal of IU1’s effect suggests potential additional effects on tumor cells or other stromal components, warranting further investigation. Second, the *in vivo* model used is immunogenic; the efficacy of this strategy in less immunogenic or metastatic CRC models needs evaluation. Third, although we focused on USP14 due to inhibitor availability, other DUBs (USP29, USP43) were also differentially expressed in TAMs, suggesting a broader regulatory network deserving future exploration. In addition, we acknowledge that the current study does not identify a direct substrate of USP14 in macrophages, and the mechanistic conclusions are therefore drawn from functional and pharmacological data. Nevertheless, the consistent involvement of MAPK signaling across multiple experimental approaches supports its role as a key downstream effector of USP14 in TAM reprogramming.

In conclusion, we have identified USP14 as a key molecular switch controlling the immunosuppressive function of TAMs in colon cancer. By demonstrating that the pharmacological inhibition of USP14 reprograms the TME, revitalizes anti-tumor T cell immunity, and synergizes with PD-1 blockade, our work provides a compelling preclinical rationale for targeting USP14 as a novel immunotherapeutic strategy. This approach offers a promising avenue to expand the fraction of patients with CRC who can benefit from immunotherapy by directly dismantling a central pillar of the suppressive TME.

### Limitations of the study

This study has several limitations. First, all *in vivo* experiments were conducted using a single, subcutaneously implanted MC38 syngeneic mouse model. While this model is immunogenic and valuable for mechanistic studies, it does not fully recapitulate the complexity of human CRC, particularly the heterogeneity of MSS tumors or the biology of orthotopic and metastatic disease. Moreover, we focused exclusively on the pharmacological inhibition of USP14 using IU1; although genetic knockdown experiments support our conclusions, future studies using conditional knockout mice (e.g., LysM-Cre mediated deletion in myeloid cells) would provide more definitive genetic evidence for the cell-specific role of USP14 in TAMs. Second, although we identify the activation of MAPK signaling (JNK and p38) as a critical downstream effector of USP14 inhibition in macrophages, the direct ubiquitinated substrate of USP14 responsible for initiating this signaling cascade remains unidentified. Our conclusions regarding the mechanism are therefore drawn from functional, pharmacological, and transcriptomic data rather than from the identification of a specific deubiquitination target. Future unbiased proteomic and ubiquitin remnant profiling studies in macrophages will be essential to pinpoint the direct molecular link between USP14 activity and the MAPK pathway. Third, while the combination of IU1 and anti-PD-1 therapy showed robust synergy, we observed that IU1 treatment upregulated PD-L1 specifically on tumor cells, likely as an adaptive response to IFN-γ produced by activated T cells. Although this provided a strong rationale for the combination, the causal relationship between T cell-derived IFN-γ and tumor cell PD-L1 expression was not formally proven in this study through IFN-γ neutralization or the use of IFN-γ receptor-deficient tumor cells. Furthermore, we did not explore whether USP14 inhibition might upregulate additional compensatory immune checkpoints beyond PD-L1 that could limit the durability of the combination therapy. Fourth, the clinical relevance of our findings is supported by TCGA and single-cell RNA-seq data, but these analyses are correlative. The current study lacks direct validation using human primary TAMs isolated from patients with CRC to confirm that USP14 is functionally required for their M2-like phenotype. Consequently, while our data provide a strong preclinical rationale, the therapeutic efficacy and safety of targeting USP14 in human patients remain to be determined, and the potential on-target/off-tumor effects of systemic USP14 inhibition on other immune cell populations or normal tissues warrant further investigation.

## Resource availability

### Lead contact

Further information and requests for resources and reagents should be directed to and will be fulfilled by the Lead Contact, Yang Yu (13554149135@163.com).

### Materials availability

This study did not generate new unique reagents. All antibodies, chemicals, commercial kits, software, and instrumentation are listed in the key resources table.

### Data and code availability

Raw RNA-seq reads have been deposited in the NCBI GEO repository (GSE320101). No custom code was used. Additional information required to reanalyze the data reported in this paper is available from the lead contact upon request. Source data for other figures will also be provided upon request.

## Acknowledgments

This study was supported by grants from the Funding Scheme for Research Projects of 10.13039/100007880Jianghan University (No. 2023KJZXB02).

## Author contributions

Conceptualization, funding acquisition, project administration, methodology, supervision, resources, and writing-review and editing, Y.Y. and H.J.; methodology, visualization, data curation, and writing-original draft, D.X.; software and formal analysis, J.F. and H.J.; validation, Dan Xiao and Jun Fang; investigation, Y.Y. and H.J.; writing review and editing, Y.Y. and H.J.

## Declaration of interests

The authors declare no competing interests.

## Declaration of generative AI and AI-assisted technologies in the writing process

The authors declare no AI was used to generate or evaluate data or to write the manuscript.

## STAR★Methods

### Key resources table

**Table:** 

REAGENT or RESOURCE	SOURCE	IDENTIFIER
**Antibodies**		

Fc block (anti-CD16/32 antibody)	Biolegend	Cat #101320
PE/Cyanine7 anti-mouse CD45 Antibody	Biolegend	Cat #103114
Brilliant Violet 785™ anti-mouse CD45 Antibody	Biolegend	Cat #157223
PE anti-mouse CD206	Biolegend	Cat #141706
FITC anti-mouse/human CD11b Antibody	Biolegend	Cat #101205
APC/Cyanine7 anti-mouse/human CD11b Antibody	Biolegend	Cat #101225
APC anti-mouse CD86 Antibody	Biolegend	Cat #105012
PE anti-mouse CD4 Antibody	Biolegend	Cat #100408
Brilliant Violet 510™ anti-mouse CD8a Antibody	Biolegend	Cat #100752
APC anti-mouse Ly-6G/Ly-6C (Gr-1) Antibody	Biolegend	Cat #108411
PE anti-mouse CD274 (B7-H1, PD-L1) Antibody	Biolegend	Cat #124308
PE/Cyanine7 anti-mouse F4/80 Antibody	Biolegend	Cat #123113
Brilliant Violet 650™ anti-mouse CD11c Antibody	Biolegend	Cat #117339
APC anti-mouse CD326 (Ep-CAM) Antibody	Biolegend	Cat #118213
PE anti-mouse IFN-γ Antibody	Biolegend	Cat #505808
Alexa Fluor® 647 anti-mouse Ly-6G Antibody	Biolegend	Cat #127609
APC/Cyanine7 anti-mouse I-Ab Antibody	Biolegend	Cat #116425
Alexa Fluor® 647 anti-mouse FOXP3 Antibody	Biolegend	Cat #126407
Alexa Fluor® 647 anti-TCF1 (TCF7) Antibody	Biolegend	Cat #655203
PE anti-mouse CD69 Antibody	Biolegend	Cat #104507
Anti-USP14	Cell Signaling Technology	Cat #4879
Anti-GAPDH	Servicebio	Cat #GB11002
HRP Goat anti-Rabbit IgG (H+L)	Abclonal, China	Cat #AS014
JNK	Cell Signaling Technology	Cat #9252
p-JNK	Cell Signaling Technology	Cat #4668
ERK1/2	Cell Signaling Technology	Cat #4695
P-ERK1/2	Cell Signaling Technology	Cat #4370
P38	Cell Signaling Technology	Cat #8690
p-P38	Cell Signaling Technology	Cat #4511

**Chemicals, peptides, and recombinant proteins**		

DAPI	Servicebio	Cat#G1012
DMEM	Gibco™, USA	12100061
FBS	Gibco, USA	A5670701
Recombinant Trypsin	Beyotime, China	P4209
Trypsin-EDTA, 0.25%	Solarbio, China	T1300
4% paraformaldehyde	Beyotime	Cat#P0099
TRIzol reagent	Invitrogen	Cat#15596018
IU1	MedChemExpress	HY-13817
SB203580	MedChemExpress	HY-10256
SP600125	MedChemExpress	HY-12041
U0126-EtOH	MedChemExpress	HY-12031
Recombinant Mouse IL-4 (carrier-free)	Biolegend	Cat#574304
Recombinant Mouse IL-13 (carrier-free)	Biolegend	Cat#575904
Recombinant Mouse M-CSF (carrier-free)	Biolegend	Cat#576404
CFDA SE	Biosharp	BL919A
LPS	Biosharp	BS464
DMSO	Biosharp	BL165B
Tween-80	Biosharp	BS118
PEG300	Aladdin	P615502
Anti-PD-1 antibody	Invitrogen	MA5-48236
Clodronate liposomes	YEASEN	40337ES08

**Critical commercial assays**		

MicroElute Total RNA Kit R6831-01	Omega Bio-tek	R6831-01
HiScript III RT SuperMix (+ gDNA wiper)	Vazyme, China	R323
The AceQ® Universal SYBR qPCR Master Mix	Vazyme, China	Q513-02/03
CCK8 assay kit	Biosharp	BL132B
Opal™ 7-Color Manual IHC Kit	Akoya Biosciences	NEL861001KT
Zombie NIR™ Fixable Viability Kit	Biolegend	Cat#423106
LEGENDplexTM Multi-Analyte Flow Assay Kit	Biolegend	Cat# 740750
BCA Protein Assay Kit	Beyotime	Cat# P0012
RIPA lysis buffer	Beyotime	Cat# P0013B
ECL kit	NCM Biotech	Cat# P10100
Lipofectamine RNAiMAX	Invitrogen	Cat#13778100
PCR-based method for mycoplasma contamination test	Beyotime	Cat# C0301S

**Deposited data**		

Analyzed Data (RNA-seq)	This paper	GSE320101

**Experimental models: Cell lines**		

Mouse bone marrow-derived macrophages (BMDMs)	This paper	–
Mouse colon cancer cells MC38	Procell	CL-0972
Mouse macrophage ANA-1	EK-Bioscience	CC-Y2010
Tumor-Associated Macrophages (TAMs)	This paper	–

**Experimental models: Organisms/strains**		

Mouse: C57BL/6J	Slake Kingda Laboratory Animal Co	–

**Oligonucleotides**		

Primers in this paper, see STAR Methods for RNA extraction and quantitative PCR	Sangon Biotech Co., Ltd	–
USP14 siRNA Target sequence:5'-GCAUGAAGAUCCUGAUCAATT-3	Wuhan Biorbyt Biotechnology Co.,Ltd	orb1843108

**Software and algorithms**		

ImageJ	NIH	https://imagej.nih.gov/ij/
GraphPad Prism v9.0	GraphPad Software	https://www.graphpad.com/
FlowJo	FlowJo, LLC	https://www.flowjo.com/
DESeq2 (RNA-seq)	Bioconductor	https://bioconductor.org/packages/DESeq2/
Cell Ranger v8.0.1	10X Genomics	https://www.10xgenomi cs.com/support/softwar e/cellranger/downloads
Seurat v5.1.0	Hao et al., 2023	https://satijalab.org/seu rat/

### Experimental model and study participant details

#### Animals

Female C57BL/6J mice (6–8 weeks old) were purchased from Hunan Slake Kingda Laboratory Animal Co. (China). Mice were housed in specific pathogen-free facilities at 22–24°C with 40%–70% humidity and a 12 h light/dark cycle, and were provided with standard chow and water *ad libitum*. All animal experiments were approved by the Animal Experimental Committee of Jianghan University (approval number JHUN-JC-2024015) and conducted in accordance with institutional and national guidelines. For tumor implantation, 2 × 10^6^ MC38 cells in 100 μL PBS were injected subcutaneously into the right flank. Mice were randomly allocated to treatment groups (n = 6–10 per group) on day 7 after inoculation. The study used only female mice; therefore, the potential influence of sex on the results could not be assessed and remains a limitation (see Discussion).

#### Cell lines

MC38 (mouse colon adenocarcinoma) was obtained from Procell. ANA-1 (mouse macrophage) was obtained from EK-Bioscience. Both cell lines were authenticated by the suppliers using short tandem repeat (STR) profiling. Cells were maintained in DMEM supplemented with 10% fetal bovine serum and 1% penicillin/streptomycin at 37 °C with 5% CO_2_. All cell lines were routinely tested for mycoplasma contamination using a PCR-based method and were confirmed negative throughout the study.

#### Primary cell cultures

Bone marrow-derived macrophages (BMDMs): Bone marrow cells were flushed from femurs and tibiae of 6- to 8-week-old female C57BL/6J mice and cultured in DMEM supplemented with 10% FBS, 1% penicillin/streptomycin, and 20 ng/mL recombinant mouse M-CSF for 7 days to generate naïve BMDMs. For polarization, BMDMs were stimulated with 20 ng/mL IL-4 plus 20 ng/mL IL-13 (M2) or 50 ng/mL LPS (M1) for 24 h.

Tumor-associated macrophages (TAMs): TAMs were isolated from MC38 tumors 14 days after inoculation. Tumors were dissected, digested with collagenase IV (0.32 mg/mL) and hyaluronidase (0.5 mg/mL) at 37 °C for 1 h, and passed through a 70 μm strainer. Single-cell suspensions were subjected to CD11b magnetic enrichment followed by FACS sorting for live CD45^+^CD11b^+^F4/80^+^ cells. Purity of sorted TAMs was routinely >95%. All primary cell procedures were approved by the institutional animal care committee.

#### Human data

This study analyzed publicly available data from The Cancer Genome Atlas (TCGA) colon adenocarcinoma (COAD) cohort (n = 471 tumor, 41 normal) and the single-cell RNA-seq dataset GSE132465. No direct human participants or samples were involved; therefore, sample size allocation and ethical approval are not applicable.

#### Animal experiments

Female C57BL/6J mice (6-week) were purchased from Hunan Slake Kingda Laboratory Animal Co. All animal experiments were approved by the Animal Experimental Committee of the Institute of Jianghan University (JHUN-JC-2024015‌) and conducted in accordance with institutional and national guidelines for the care and use of animals. Mice were housed at 22∼24 °C and 40%∼70% humidity, and were fed and watered freely by alternating light and dark for 12 h. All animal experiments were performed in a double-blind manner. 100 μL PBS containing 2×10^6^ MC38 tumor cells was subcutaneously injected into the right flank of female C57BL/6 mice to develop the subcutaneous tumor bearing model. The mice were divided randomly into different groups seven days later. IU1 (20 mg/kg, i.p.), dissolved in a solution (10% DMSO, 5% Tween-80 and 40% PEG300 in PBS) and PBS (100 μL, i.p.) were injected every three day from day 6 to day 15, respectively. The dosing schedule (every 3 days) was based on prior pharmacokinetic studies of IU1 and our preliminary efficacy/toxicity tests[29]. No overt signs of toxicity were observed in treated mice.Clodronate liposomes were applied for macrophage depletion in the dose of 150 μL per mouse for on the first day, followed by 100 μL per mouse every three days for a total of four times. Treatments with anti-PD-1 (7.5 mg/kg, i.p.) were conducted every other day from day 7 to day 13, for a total of four times. The length (L) and width (W) of the tumors were measured using vernier calipers. Tumour volume (V) was calculated as V = (L×W^2^)/ 2.

#### Cell culture and treatments

Mouse MC38 and ANA-1 cells were obtained from ATCC. MC38 was grown in 1640 medium and ANA-1 was grown in Dulbecco’s Modified Eagle’s Medium (DMEM) (Gibco, Grand Island, NY, USA) containing 10% Fetal Bovine Serum (FBS) (Gibco, Grand Island, NY, USA) and 1% penicillin/streptomycin solution.

Mouse bone marrow-derived macrophages (BMDMs) were cultured with 20 ng/mL IL-4 /IL-13 to be polarized into M2 macrophage; or 50 ng/mL LPS to be polarized into M1 macrophage. All cytokines were purchased from PeproTech (Rocky Hill, NJ, USA). All the cells were cultured with DMEM supplemented with 10% FBS, 1% Penicillin/Streptomycin, and 20 ng/mL M-CSF (PeproTech) for differentiation.

Following the generation of single-cell suspensions from MC38 tumors as described, TAMs were positively selected or sorted for subsequent *in vitro* experiments. For RNA and protein extraction, CD11b^+^ cells were first enriched using magnetic-activated cell sorting (MACS) with anti-CD11b microbeads (Miltenyi Biotec) according to the manufacturer’s protocol. For functional assays requiring high purity, TAMs (defined as live CD45^+^CD11b^+^F4/80^+^ cells) were isolated by fluorescence-activated cell sorting (FACS) using a BD FACSAria III cell sorter (BD Biosciences). Sorted cells were collected in RPMI-1640 medium supplemented with 10% FBS for immediate culture or in appropriate lysis buffers for molecular analysis.

### Method details

#### RNA sequencing and bioinformatics analysis

For transcriptomic profiling, total RNA was extracted from FACS-sorted TAMs (CD45^+^CD11b^+^F4/80^+^) pooled from 3-4 tumors per group (PBS vs. IU1-treated) using the MicroElute Total RNA Kit. RNA integrity was verified using an Agilent 2100 Bioanalyzer. Library preparation was performed using the NEBNext® Ultra™ II Directional RNA Library Prep Kit (New England Biolabs), and sequencing was carried out on an Illumina NovaSeq 6000 platform (Novogene Co., Ltd.) to generate 150 bp paired-end reads. Raw sequencing reads were quality-controlled using FastQC and aligned to the mouse reference genome (GRCm38) using HISAT2. Gene expression quantification and differential expression analysis were performed with StringTie and Ballgown. Genes with a fold change > 2 and an adjusted P-value < 0.05 were considered significantly differentially expressed. Functional enrichment analysis of Kyoto Encyclopedia of Genes and Genomes (KEGG) pathways was conducted using the clusterProfiler R package.

#### Multiplex immunofluorescence staining and quantitative analysis

To visualize and quantify specific cell populations within the tumor architecture, formalin-fixed, paraffin-embedded (FFPE) tumor sections (5 μm thickness) were used. After deparaffinization and antigen retrieval, multiplex immunofluorescence staining was performed using the Opal™ 7-Color Manual IHC Kit (Akoya Biosciences) according to the manufacturer's instructions. The following primary antibody and Opal fluorophore combinations were used: anti-F4/80 (Opal 520), anti-CD206 (Opal 570), and anti-CD8a (Opal 690). Nuclei were counterstained with DAPI. Stained slides were scanned using the Vectra Polaris Automated Quantitative Pathology Imaging System (Akoya Biosciences) at 20x magnification. Image analysis and spectral unmixing were performed using the inForm® software (Akoya Biosciences).

#### Analysis of cell-type-specific PD-L1 expression by flow cytometry

To evaluate PD-L1 expression on different cell populations within the tumor microenvironment, the single-cell suspensions prepared for general flow cytometry were simultaneously stained with the following antibody panel: Zombie NIR™ Fixable Viability Kit, anti-CD45 (BV785), anti-CD11b (APC-Cy7), anti-F4/80 (PE-Cy7), anti-CD11c (BV650), anti-CD326 (EpCAM, APC) for tumor cells, and anti-PD-L1 (PE). Fluorescence-minus-one (FMO) controls were used for gating. PD-L1 geometric mean fluorescence intensity (gMFI) was analyzed separately on gated populations: tumor cells (CD45^-^), dendritic cells (CD45^+^CD11c^+^), and macrophages (CD45^+^CD11b^+^F4/80^+^).

#### Real-time quantitative PCR

Total RNA was harvested from cells using MicroElute Total RNA Kit R6831-01 (Omega Bio-tek, Norcross, GA, USA) and reverse-transcribed into cDNA using HiScript III RT SuperMix (+ gDNA wiper) (Vazyme, Nanjing, China). The cDNA was amplified using the AceQ® Universal SYBR qPCR Master Mix (Vazyme, Nanjing, China) on a StepOnePlus Real-Time PCR System (Thermo Fisher Scientific, Waltham, MA, USA). Primer sequences were obtained by referring to previous studies. All primers were synthesized by Sangon Biotech Co., Ltd (Shanghai). All the primer used in the experiment were showed in supporting information Table S1.

#### Transfections

BMDMs were seeded in 6-well-plates (1×10^5^ cells per well). After 24 h, they were transfected with either small interfering RNA (siRNA) against USP14 or negative control (NC) siRNA using Lipofectamine RNAiMAX (Invitrogen, Carlsbad, CA, USA). Cells were harvested after 48 h and processed for western blotting and flow cytometry. USP14 siRNA (orb1843108, Target sequence: 5'-GCAUGAAGAUCCUGAUCAATT-3) was purchased from Wuhan Biorbyt Biotechnology Co.,Ltd.

#### Western blotting

Lysing cells with RIPA lysis buffer (with protease and phosphatase inhibitors), and protein quantification with BCA assay, followed by SDS-PAGE separation and transfer to PVDF membrane. After blocking the membranes for 1 h at room temperature in 5% skim milk powder dissolved in Tris-buffered saline containing 5% Tween-20 (TBST), membranes were incubated overnight at 4 °C with the corresponding antibodies and membrane wash conditions (TBST, 3 × 10 min). Then, membranes were washed and incubated with secondary antibodies prior to detection. They were developed using NcmECL Ultra (P10100, NCM Biotech) and imaged. All the antibody used in the experiment were showed in supporting information Table S2.

#### Flow cytometry

MC38 tumors from mice were digested into single cell by cutting into small pieces and incubating with Collagenase IV (0.32 mg /mL) and hyaluronidase (0.5 mg /mL) for 1 h at 37 °C. The tumor cells were filtered through 70 μm cell strainer after lysis of RBCs. All the samples were infused with Fc block (anti-CD16/32 antibody, clone 93, a 1:100 dilution) followed by Zombie NIR™ Fixable Viability Kit (423106), CD45 (103114), CD11b (101205), CD86 (105012), CD4 (100408), CD8a (100752), Gr-1 (108411), and PD-L1 (124308). For the T-cell intracellular IFN-γ (505808) cytokine staining, cells were fixed and permeabilized after stimulation with Phorbol 12-myristate 13-acetate (PMA) (ab120297, Abcam, 100 ng/mL), Monensin sodium salt (ab120499, Abcam, 1 ug/mL), and Ionomycin calcium salt (5608212, PeproTech, 100 ng/mL) for 6 h. For the CD206 (141706) staining, cells were also fixed and permeabilized, and then measured via fow cytometry.

#### Tissue cytokine detection

Cytokines IFN-γ, TNF-α, IL-2, IL-4, IL-12 and IL-6 in tumor tissues extracted from either the Control group or the DUB-IN-1 group were detected by flow cytometry according to LEGENDplexTM Multi-Analyte Flow Assay Kit (740750, Biolegend).

### Quantification and statistical analysis

Statistical analysis was performed using GraphPad Prism 6.0 software. One-way ANOVA with Tukey’s multiple comparisons test was used to compare three or more groups. A two-tailed unpaired t-test or the Mann-Whitney U test was used to compare two groups. P-values<0.05 were considered statistically significant. Data are presented as means ± standard error of the mean (SEM). ∗P < 0.05, ∗∗P < 0.01, ∗∗∗P < 0.001, NS: not significant.

## References

[1] Siegel R.L., Wagle N.S., Cercek A., Smith R.A., Jemal A. (2023). Colorectal cancer statistics, 2023. CA Cancer J. Clin..

[2] Cervantes A., Adam R., Roselló S., Arnold D., Normanno N., Taïeb J., Seligmann J., De Baere T., Osterlund P., Yoshino T. (2023). Metastatic colorectal cancer: ESMO Clinical Practice Guideline for diagnosis, treatment and follow-up. Ann. Oncol..

[3] Audisio A., Fazio R., Daprà V., Assaf I., Hendlisz A., Sclafani F. (2024). Neoadjuvant chemotherapy for early-stage colon cancer. Cancer Treat Rev..

[4] Roelands J., Kuppen P.J.K., Ahmed E.I., Mall R., Masoodi T., Singh P., Monaco G., Raynaud C., de Miranda N.F.C.C., Ferraro L. (2023). An integrated tumor, immune and microbiome atlas of colon cancer. Nat. Med..

[5] Angell H.K., Bruni D., Barrett J.C., Herbst R., Galon J. (2020). The Immunoscore: Colon Cancer and Beyond. Clin. Cancer Res..

[6] Ciardiello F., Ciardiello D., Martini G., Napolitano S., Tabernero J., Cervantes A. (2022). Clinical management of metastatic colorectal cancer in the era of precision medicine. CA Cancer J. Clin..

[7] Guo L., Wang Y., Yang W., Wang C., Guo T., Yang J., Shao Z., Cai G., Cai S., Zhang L. (2023). Molecular Profiling Provides Clinical Insights Into Targeted and Immunotherapies as Well as Colorectal Cancer Prognosis. Gastroenterology.

[8] Kraehenbuehl L., Weng C.H., Eghbali S., Wolchok J.D., Merghoub T. (2022). Enhancing immunotherapy in cancer by targeting emerging immunomodulatory pathways. Nat. Rev. Clin. Oncol..

[9] Li Y., Zhou H., Liu P., Lv D., Shi Y., Tang B., Xu J., Zhong T., Xu W., Zhang J. (2023). SHP2 deneddylation mediates tumor immunosuppression in colon cancer via the CD47/SIRPα axis. J. Clin. Investig..

[10] Sathe A., Mason K., Grimes S.M., Zhou Z., Lau B.T., Bai X., Su A., Tan X., Lee H., Suarez C.J. (2023). Colorectal Cancer Metastases in the Liver Establish Immunosuppressive Spatial Networking between Tumor-Associated SPP1+ Macrophages and Fibroblasts. Clin. Cancer Res..

[11] Zhang L., Li Z., Skrzypczynska K.M., Fang Q., Zhang W., O'Brien S.A., He Y., Wang L., Zhang Q., Kim A. (2020). Single-Cell Analyses Inform Mechanisms of Myeloid-Targeted Therapies in Colon Cancer. Cell.

[12] Wang L., Li S., Luo H., Lu Q., Yu S. (2022). PCSK9 promotes the progression and metastasis of colon cancer cells through regulation of EMT and PI3K/AKT signaling in tumor cells and phenotypic polarization of macrophages. J. Exp. Clin. Cancer Res..

[13] Blériot C., Chakarov S., Ginhoux F. (2020). Determinants of Resident Tissue Macrophage Identity and Function. Immunity.

[14] Chen D., Xie J., Fiskesund R., Dong W., Liang X., Lv J., Jin X., Liu J., Mo S., Zhang T. (2018). Chloroquine modulates antitumor immune response by resetting tumor-associated macrophages toward M1 phenotype. Nat. Commun..

[15] Saeed M., Gao J., Shi Y., Lammers T., Yu H. (2019). Engineering Nanoparticles to Reprogram the Tumor Immune Microenvironment for Improved Cancer Immunotherapy. Theranostics.

[16] Perkins H., Khodai T., Mechiche H., Colman P., Burden F., Laxton C., Horscroft N., Corey T., Rodrigues D., Rawal J. (2012). Therapy with TLR7 agonists induces lymphopenia: correlating pharmacology to mechanism in a mouse model. J. Clin. Immunol..

[17] Xia X., Huang C., Liao Y., Liu Y., He J., Guo Z., Jiang L., Wang X., Liu J., Huang H. (2019). Inhibition of USP14 enhances the sensitivity of breast cancer to enzalutamide. J. Exp. Clin. Cancer Res..

[18] Ma A., Tang M., Zhang L., Wang B., Yang Z., Liu Y., Xu G., Wu L., Jing T., Xu X. (2019). USP1 inhibition destabilizes KPNA2 and suppresses breast cancer metastasis. Oncogene.

[19] Guo F., Zhang C., Wang F., Zhang W., Shi X., Zhu Y., Fang Z., Yang B., Sun Y. (2020). Deubiquitinating enzyme USP33 restrains docetaxel-induced apoptosis via stabilising the phosphatase DUSP1 in prostate cancer. Cell Death Differ..

[20] Lai C.Y., Yeh D.W., Lu C.H., Liu Y.L., Chuang Y.C., Ruan J.W., Kao C.Y., Huang L.R., Chuang T.H. (2020). Epigenetic Silencing of Ubiquitin Specific Protease 4 by Snail1 Contributes to Macrophage-Dependent Inflammation and Therapeutic Resistance in Lung Cancer. Cancers (Basel).

[21] Gutierrez-Diaz B.T., Gu W., Ntziachristos P. (2020). Deubiquitinases: Pro-oncogenic Activity and Therapeutic Targeting in Blood Malignancies. Trends Immunol..

[22] Lei C.-Q., Wu X., Zhong X., Jiang L., Zhong B., Shu H.B. (2019). USP19 Inhibits TNF-α–and IL-1β–Triggered NF-κB Activation by Deubiquitinating TAK1. J. Immunol..

[23] Li L., Wei J., Li S., Jacko A.M., Weathington N.M., Mallampalli R.K., Zhao J., Zhao Y. (2019). The deubiquitinase USP13 stabilizes the anti-inflammatory receptor IL-1R8/Sigirr to suppress lung inflammation. EBioMedicine.

[24] Shi D., Wu X., Jian Y., Wang J., Huang C., Mo S., Li Y., Li F., Zhang C., Zhang D. (2022). USP14 promotes tryptophan metabolism and immune suppression by stabilizing IDO1 in colorectal cancer. Nat. Commun..

[25] Dai X., Lu L., Deng S., Meng J., Wan C., Huang J., Sun Y., Hu Y., Wu B., Wu G. (2020). USP7 targeting modulates anti-tumor immune response by reprogramming Tumor-associated Macrophages in Lung Cancer. Theranostics.

[26] Zhao C., Gong J., Bai Y., Yin T., Zhou M., Pan S., Liu Y., Gao Y., Zhang Z., Shi Y. (2023). A self-amplifying USP14-TAZ loop drives the progression and liver metastasis of pancreatic ductal adenocarcinoma. Cell Death Differ..

[27] Morgan E.L., Toni T., Viswanathan R., Robbins Y., Yang X., Cheng H., Gunti S., Huynh A., Sowers A.L., Mitchell J.B. (2023). Inhibition of USP14 promotes TNFα-induced cell death in head and neck squamous cell carcinoma (HNSCC). Cell Death Differ..

[28] Zou W. (2005). Immunosuppressive networks in the tumour environment and their therapeutic relevance. Nat. Rev. Cancer.

[29] Lee B.H., Lee M.J., Park S., Oh D.C., Elsasser S., Chen P.C., Gartner C., Dimova N., Hanna J., Gygi S.P. (2010). Enhancement of proteasome activity by a small-molecule inhibitor of USP14. Nature.

[30] Lee B.H., Lu Y., Prado M.A., Shi Y., Tian G., Sun S., Elsasser S., Gygi S.P., King R.W., Finley D. (2016). USP14 deubiquitinates proteasome-bound substrates that are ubiquitinated at multiple sites. Nature.

[31] Moon S., Muniyappan S., Lee S.B., Lee B.H. (2021). Small-Molecule Inhibitors Targeting Proteasome-Associated Deubiquitinases. Int. J. Mol. Sci..

[32] Lee H.O., Hong Y., Etlioglu H.E., Cho Y.B., Pomella V., Van den Bosch B., Vanhecke J., Verbandt S., Hong H., Min J.W. (2020). Lineage-dependent gene expression programs influence the immune landscape of colorectal cancer. Nat. Genet..

[33] Gubat J., Sjöstrand L., Selvaraju K., Telli K., D'Arcy P. (2024). Loss of the proteasomal deubiquitinase USP14 induces growth defects and a senescence phenotype in colorectal cancer cells. Sci. Rep..

[34] DeNardo D.G., Ruffell B. (2019). Macrophages as regulators of tumour immunity and immunotherapy. Nat. Rev. Immunol..

[35] Fraile J.M., Quesada V., Rodríguez D., Freije J.M.P., López-Otín C. (2012). Deubiquitinases in cancer: new functions and therapeutic options. Oncogene.

