# Supplementary material for: Targeting USP14 enhances immunotherapy response by reprogramming tumor-associated macrophages in colon cancer
Source: iScience. 2026 Mar 13;29(5):115362. doi: 10.1016/j.isci.2026.115362 (PMC13122227; doi:10.1016/j.isci.2026.115362)
Supplement: Document S1. Tables S1 and S2 [file mmc1.pdf]

iScience, Volume *29*

Supplemental information

Targeting USP14 enhances immunotherapy response by reprogramming tumor-associated macrophages in colon cancer

Dan Xiao, Jun Fang, Hui Jian, and Yang Yu

Table S1. RT-PCR primer information

Gene	Forward primer	Reverse primer
UCHL1	TCTGTCTGAAACGGAGAAGCTGTC	CATGGTTCACTGGAAAGGGCATTC
UCHL3	AGACCAGTATGCGCAGTGTT	TCCAATCGTTCCACAGGCAT
UCHL5	ACAACTTGCAGAGGAACCCAT	CTGGTGCTCAGCTAAGGTCT
BAP1	GAGAGGACGAGGAGTGGACAGATA	CTGCCATCAGGTTGAAGCGAATG
JOSD1	AGACAAGGCCAAATCTGAATCCTT	ATGCTCTTCTTGTGGGGTGTT
JOSD2	GAAGCGAGACCAAGATCACCC	GGGCTAGCCTCTTGCAGATT
ATAXIN3	GTGCTCAGCATTGCCTGAATAACC	TTCTCAGCCTCTCCTCTTCATCCA
OTUD1	CGATTCCACATCATCCCCGA	CCCTCAATGAGTGGGCTGAA
OTUD3	CTTCGTGGAAGATGACATTCCC	AAAGGGGCATTAAGCTGATGG
OTUD4	AGGCAGACATAGACTTGGCTTCAG	TGGTCCTCTTAGGCTCAGGTTCA
OTUD5	AGGACGGTGCCTGTCTATTTC	CATCAGATAGTCCATGCAATGCT
OTUD6A	GCCAACTCACCCACCATTGTCA	CCGAATTGTAGTGCTCTCCGAAGT
OTUD6B	GCAGCTCACGGAAGATGTTG	AATCCGAGGTGGTTGATTCTCA
OTUD7A	ACAGCAGAACAAGGAGGAGGAATG	AGTATGTGGGCGAGGACAAAGAC
OTUD7B	CGGAAGATGAGTGGCAGAAGGAA	TGAGCAAGGACGAAGACATGGAAT
OTUDB1	GCTGTGCAGAATCCTCTGGT	AAGCCAAACGCTCGGTAGAA
OTUDB2	AACTCAGCAAAAGATTCACCTCG	TCATTTGGGGTCTGTAGCACA
YOD1	GCGATACTGGGAAAGACAAACG	AGCGGATCGTAGTGAATGCC
OTULIN	ACGATGACAAGGAGAAGGGAAAGG	CGGACAGCATAGGCAAGAAGGAA
TRABID	TGTGTGGGTGTTGTGGAAGG	GCGTATGGCCAGATGTACCA
VCPIP1	AACCAGAGCACGTCAACACA	CATGCACTAGGCAGTGTCCA
PSMD7	GGACGCCAACGTCAAAAACT	AGTCCCCACTGTAGTGTCCT
PSMD14	AAACATGGTCGTGCTGGAGT	CCAGTTCCTGACTGTGGCAT
AMSH	CCCTGAAACCTGGAGCGTTA	AGGTTTCAATGCCTTTGGCG
PRPF8	TGGCCGGAGTGTTTCCTTAC	CATATCGCTTGGCCTGCAAC
BRCC3	GCAGGCGGTTCATCTTGAGT	GACTGTGCGCATTTCCGTTC
EIF3H	CACACGGAGGATGATGCTGACTT	TGAGGACGACAGACTCTTCAATGG
EIF3F	CACCAACTGCTTTTCGGTGC	TGTCATGGCCTGTGGCATAC
MYSM1	TTCTAATAGCAGCGTCCCCA	ACTCCTCAGCGATGAAGCAA
COPS6	GCCTATGCCCTATGTCACTGTCTT	CCAATGCCTTGTCGGTCGTAGA
MPND	AAGAAGATGTGCTGGCTGGG	TTGTCCATACGCTTCCCTGG
USP1	TAATGGGCTTTCAAGAGGCAGT	TCTTCTCGCAGCTTACTGGG
USP2	GTGCTCCACCTGAAGCGATTCT	GGACTTCGGCAGTAGGCTGTATAG
USP3	GTTTGCTCCTTAAGAGATTGCC	TCTCAGTGGGAATTGAACGTAT
USP4	CCAAAAGGTGAAAGGCCAGC	TGCTCAGGTGCTTCTCACAG
USP5	GAAGACGGACAAGACGATGACTGA	ACCTGGACCACGGAGTTGAGATA
USP7	TCGTCGCACATTGAGACGG	CTTGTCGGCATGGTTGGGAAT
USP8	GCCTGCTGTAGCTTCAGTTC	AAGATCTTCTGCGCGCTGTG
USP9X	GACCGGGACACCCAAAAGAT	CATGGGGACTTCGCTGACTT
USP9Y	AGTTTGCTGTGAAGCCCTGGAA	AGAACTGCTCCTGTGCCAACTG

USP10	CTGAAGCCGTTGAAAAAGATGAG	TCAGCCTCTGCGTTAGAGTTG
USP11	GCAGAACCATAAACGACGAAAT	CACAGATCTGAGATATTGCCCT
USP12	CAGTCTCCAAATTCGCCTCCA	GTGCTCGTTGACCGGAAACT
USP13	CAGTCTGCCCTTGGCATTTAC	CCTCTTCTCTTAGGGAGCACAG
USP14	ACCTCCAATGGTGTTCAAAGC	CATCCTTCAGGGTTCCTCCTT
USP15	TCAGCCATTCAGTGTTTGAGC	TTTCACCTCTCATTCCTAAGGGA
USP16	TGGCTCCTTTTTGTACCCTTAA	GTACCACTGTGTTCAACAACTC
USP18	CAGGAGTCCCTGATTTGCGTG	CAGAGGCTTTGCGTCCTTATC
USP19	TGGAGATGCTAGGAGAGTGTC	CGCAGCTTAACAATCACCTCAT
USP20	TGGACTGCATAGGGGAGGTG	GGCAAGCCCATAGGTTAGGTC
USP21	AACTCCATGTTACGACCTTTGC	AAGGGGACCTCTAGGACGAGA
USP22	TCTTTCTGTCGGATAGGCACC	GCCCTGAGTAAAACTCCTGGA
USP24	GCTGGAAAGCCGCGTTTTG	CAAGTCTGGCTAAGTAGGTGGA
USP25	TCTCGAAACCCCTATGACAGAA	TGAATAACTGCACTAAACCAGCA
USP27X	GACATTGAGCAAATTGCCAAAGA	AAGTCCCGGCACTGAACAC
USP28	GGGTCCGAGAAGGAAAGCC	CACGGAACGATCCGAAGGAAG
USP29	GCTGGGTGATAATGTTACAGGC	TGAGTAAGGATGTGTCGTCTCT
USP30	CCTCACCCCACGACCAATC	GAGAAAGGCTGTCAAAGGTGT
USP31	TTCTTTGCCAATTCCTCTACCC	CACAGCCACACCGATCCTC
USP32	GTCCCAGATACACTCAGGAAGT	AGCGAGAGAAGGTAAAAGCATC
USP33	GGACCACAGCACCATACACTC	GCTGCAAGCATAACACCATACT
USP34	CTGGTTGCCTATGAAGGCTTG	AGCTTGATGCAGTTTTTCGACA
USP36	CCAACAGCGGCAATGCTATC	CATCGCATCAATGGTGTACCG
USP37	AGTCAGCCTGCTCGTTCACTA	AAGTCAACATTAGGCGGCTTT
USP38	GCCCCTCAAGCGGATGATT	GGGTCGTCAGGTCAAACATGG
USP39	GTCACTGCCCGTACTTGGATA	GTATGCGTTGATGTGCGAGAG
USP40	TGACCGACTGGTTAAAGCAGC	GCTAGTATCCTTGTAGCGTTCAC
USP42	AGGCGGTCTCACCTGAAGA	CACTGGCCCTAATGGAAGTGT
USP45	ATGCGGGTAAAAGATCCATCAAA	ACGTTAGACCTACAGCAATGTCA
USP46	ATGACTGTCCGAAACATCGCC	TTGACCAATCCGAAGTAGTGTTC
USP47	GATGTGATTCCCTTGGATTGCT	AACCCCATTGGTGTATCTTCTTC
USP48	CAGAGGAAACCCGAATTGCTT	GTGGCTCCCAGGTTAGTCAAG
USP49	AGTTCCGGGAATGTTTCCTGA	CTCCTTACTGACAACTCTGCG
USP26	ACCAATGAGGACCACAACCA	CACTCTGGTTCACACCCCTT
USP35	GGAGTGGGTTCTAAGGCAAGAA	GTAGGACGAAGTCACCACGG
USP43	GCAGGCCCGAGGTTCATC	TGTGAGGTGCCAGCTGTACT
USP44	CCGTGCCTGGTTACTGACAT	CTGCTTCTGGGCTTCTGTGA
USP50	CCTCTGCAGCGTATCTCCAC	TCATCAGGTAGGCAAAGGCG
USP51	TGCGGATGATCTATCAGCGTTTCA	CACAGGACAGACAAGAGTGGAGTC
USP52	GGCTTCCTCTTCCACATGCT	AGAAACTGCCTCCAGCACC
USP53	CTGTTACACCAGCCGACACT	AAGGGGCTGTAAGTGGCATC
USP54	GCAGAATCAGGGGAGGAGTG	TTACACAGAGCCAGAGCTGC
CYLD	ACCCTACTGGGAAGAACGGAT	CGGTCTTGGATGTACTGTCCTAT

USP17le	GGTCTTTGGAGACATGGTGGT	TCTGGGGCATCAGGAGATGA
USPL1	CAGGGACTGATGTAGGGATATCTT	GTGACTCAGAGCCCAAAGGAT
DESI2	AGATTCCTCGCTGGATCAACCG	GGCTGCATCCTCTGCTTCTTCT
Arg1	GGTTCTGGGAGGCCTATCTT	CACCTCCTCTGCTGTCTTCC
IFN-γ	GAGCCAGATTATCTCTTTCTACCT	GTTGTTGACCTCAAACTTGGC
IL-1	ACCCCAAAAGATGAAGGGCTG	TACTGCCTGCCTGAAGCTCT
IL-10	CAGAGAAGCATGGCCCAGA	TGCTCCACTGCCTTGCTCTTA
IL-12	GGAACTACACAAGAACGAGAG	AAGTCCTCATAGATGCTACCA
IL-6	GAGGATACCACTCCCAACAGACC	AAGTGCATCATCGTTGTTCATACA
TNF-α	CATCTTCTCAAAATTCGAGTGACAA	TGGGAGTAGACAAGGTACAACCC
Nos2	CACCAAGCTGAACTTGAGCG	CCATAGGAAAAGACTGCACCG

Table S2. The antibodies information

Antibody	Catalog number	Company
USP14	#4879	Cell Signaling Technology
JNK	#9252	Cell Signaling Technology
p-JNK	#4668	Cell Signaling Technology
ERK1/2	#4695	Cell Signaling Technology
P-ERK1/2	#4370	Cell Signaling Technology
P38	#8690	Cell Signaling Technology
p-P38	#4511	Cell Signaling Technology
GAPDH	GB11002	Servicebio
